# Prevalence of Gender Disparity in Professional Societies of Family Medicine: A Global Perspective

**DOI:** 10.7759/cureus.7917

**Published:** 2020-05-01

**Authors:** Aven Sidhu, Sabeena Jalal, Faisal Khosa

**Affiliations:** 1 Epidemiology and Public Health, Fraser Health Authority, Surrey, CAN; 2 Radiology, Vancouver General Hospital, Vancouver, CAN

**Keywords:** women in family medicine, primary care, gender disparity, family medicine

## Abstract

Introduction

Gender disparity in academic and organizational settings has been the topic of numerous studies, which have attributed under representation of females within medicine to both individual and institutional reasons. The main objective of our study was to assess gender disparity in leadership positions in committees of professional societies of family medicine (FM).

Methods

In this cross-sectional observational study, we collected publicly available information from 3 major FM societies (College of Family Physicians of Canada, the Royal Australian College of General Practitioners, and the World Organization of Family Doctors) and also collected the academic/leadership information for each committee member, including bibliometric parameters of their research productivity.

Results

In total, our sample size was 960 and composed of 58% men (556) and 42% women (404). There was a significant difference found in all the research productivity variables. Men had a greater number of publications, number of citations, years since first publication, years of active research, and had a larger h-index.

Conclusion

In conclusion, gender disparity within FM societies is less significant compared to other professional medical societies and creating an environment that supports women and supports research can aid in achieving gender parity.

## Introduction

Gender disparity is a phenomenon that has been studied in varying contexts from high school to post-graduate studies and further into the medical schools and professional careers [1-2]. This disparity has been documented in medical and surgical specialties, editorial boards of medical journals, and professional societies [3-4]. The under-representation of females can be attributed to both individual and institutional level reasons [2]. This results in disadvantages faced by women: perceived competence, starting wage salary, and slower promotion rate [2].

Compared to Science, Technology, Engineering, and Math (STEM) careers, medicine is a field in which women have almost attained parity with men in the ratio of matriculation and training [1]. For example, the Association of American Medical Colleges (AAMC) has recorded an increase in the proportion of women graduating from medical school from 8.9% in 1965 to 47.5% in 2014 [5]. Similarly, in Canada, the percentage of female physicians has also increased from 28.4% to 45.9% from 2000 to 2018 [6]. However, even though the number of female physicians in training is roughly equal to that of male physicians, the ratio is not maintained when advancing into further specializations, such as radiology, neurosurgery, cardiology, and even specializations that are usually considered to have a substantial number of female physicians such as dermatology [5].

The increase in female representation is also lacking in higher academic ranks and leadership positions. In the United States, while the number of female graduates has increased, only 43% of Associate Professor, and 31% of Professor positions are occupied by females [5]. Despite the efforts made in the past decades to train more female physicians, women do not advance in parity with men in academic medicine [4,7-8].

Family Medicine (FM) associations and societies are professional organizations responsible for establishing training standards, continued education, and professional development for family physicians. The ratio and ranks of male and female committee members can arguably impact the future physicians in terms of mentorship and role models. FM, as a field of medicine, has an almost equal ratio of male to female physicians in many Western countries [5,6]. Female physicians comprise 45.9% of all Canadian FM physicians, compared to 29.3% in the surgical specialties [6]. Similarly, Australia has seen an increase in the percentage of female family physicians from 36.5% to 42.1% from 2005 to 2015 [9].

Since research productivity is an important factor in the promotion to academic and leadership ranks, several factors which serve as measures of academic achievements, such as the H-index, FM society committee rank, university academic rank, publications, citations and years of active research, were measured and analyzed in our study. The H-index has become a widely used tool to numerically measure the impact of an author’s academic work [10-11]. It is generated by taking into consideration the number of publications, the number of times a publication is cited, and the overall research productivity [12]. Our study specifically examined the ratio of female and male FM physicians holding committee positions in FM societies and committee member’s scholarly productivity.

## Materials and methods

An Institutional Review Board approval was not needed for our retrospective study since we evaluated data, which is publicly available. Our methodology has been validated in several of our recent manuscripts [10-11,13]. This cross-sectional descriptive study aimed to identify the impact of research productivity on the promotion at academic and leadership ranks by retrospectively collecting information of committee members in the College of Family Physicians in Canada (CFPC), Royal Australian College of General Practitioners (RACGP) and the World Organisation of Family Doctors (WONCA). These three were selected due to the availability of information. With 118 member organizations in 131 countries, WONCA represents FM physicians from both developing and industrialized countries. Other FM societies, such as the American Board of Family Medicine (ABFM) and the Royal New Zealand College of General Practitioners (RNZCGP), were screened but not selected, as member information is not publicly available for these.

Active members of each FM Society with, both, a medical degree (MD or DO) and gender information were collected for this study. Gender was confirmed by a combination of publicly available photos and the pronouns used to address each individual in related news articles. Additional resources such as LinkedIn, Google, and institutional websites were subsequently consulted to complement and supplement any missing information. Leadership rank was defined as the position each member held within the FM society, such as President or Vice President. Academic rank was the faculty position the member held at their academic institution. Since universities in different countries may have different faculty titles, all academic ranks were compared and ranks of similar levels were grouped together (Table 1).

Data were analyzed using STATA V.14.2 to examine the correlation between gender, research productivity, and academic leadership of FM physicians. Analysis of research productivity was done by using variables such as publications, H-index, number of citations, and years since first publication. These variables were then further analyzed by leadership and academic rank. The research productivity of each society was also compared to one another.

Due to the categorical nature of the FM association’s outcome variable, multivariable analysis was performed by using ordinal logistic regression. Each variable was entered into multivariable linear regression. Univariate analysis was performed using χ2 and Wilcoxson rank-sum tests where appropriate. Generalized ordered logistic regression was used to estimate the impact of the primary predictors, including gender, leadership ranks, academic ranks, publications, citations, H-index, years since first publication, years of active research, on becoming a president in CFPC, RACGP, and WONCA.

## Results

A total of 1726 committee members were identified in our selected FM societies. After excluding non-medical doctors and missing gender information, our sample was 960; of which 556 were male (58%) and 404 were female (42%). Table 1 summarises the number of males and females by leadership rank and academic rank. There were more males than females in FM Society committees. When analyzing research productivity, males had higher median values for all research-related variables (Table 2).

**Table 1:** Leadership and academic rank by gender * or equivalent in country

		Males n (%)	Females n %)	Total (%)	p-value
Leadership Rank					0.98
1	President/Chair (First in Command)	81 (8.44%)	59 (6.15%)	140 (14.58%)	
2	Vice President/Vice Chair (Second in Command)	14 (1.46%)	11 (1.15%)	25 (2.60%)	
3	Member of committee (Only member)	461 (48.02%)	334 (34.79%)	795 (82.81%)	
Total		556 (57.92%)	404 (42.08%)	960 (100%)	
Academic Rank					0.012
1	Professor*	260 (27.08%)	199 (12.39%)	459 (47.81%)	
2	Associate Professor*	18 (1.56%)	9 (0.94%)	27 (2.81%)	
3	Assistant Professor*	32 (3.33%)	44 (4.58%)	76 (7.92)	
4	Tutor/Instructor*	65 (6.77%)	51 (5.31%)	116 (12.08%)	
5	University affiliation found – faculty with no academic rank	78 (8.12%)	50 (5.21%)	128 (13.33%)	
6	No University affiliation found	103 (10.73)	51 (5.31%)	154 (16.04%)	
Total		556 (57.92%)	404 (42.08%)	960 (100%)	

**Table 2:** Median values of variables by gender All values are median (interquartile range) unless otherwise indicated. IQR, interquartile range

Variable	Male Median (IQR)	Female Median (IQR)	p value
Publications	7 (1-629)	3 (1-220)	0.0001
H-index	4 (0-79)	3 (0-29)	0.022
Citations	70 (0-31348)	35 (0-4886)	0.0045
Years since first publication	18 (1-67)	13 (1-38)	0.0001
Years of active research	15 (1-67)	10 (1-38)	0.001

Variables by leadership and academic ranks

Overall, there were more males in all leadership ranks of FM societies compared to women (Chi-square = 0.0635; *p *= 0.98); see Table 1. When analyzing specific variables across leadership ranks, males held higher medians for publication across all leadership ranks. Males also held a higher H-index for ‘first in command’ and for ‘member’ ranks; females held higher H-index at the ‘second in command’ rank. Females had more citations in ‘first and second in command’ ranks (Table 3).

**Table 3:** Variables across leadership rank IQR, interquartile range

Variable	Leadership Rank	Male Median (IQR)	Female Median (IQR)
Publications	President	8 (1-25)	5 (1-17)
	Vice President	10 (2-182)	6 (1-12)
	Members	7 (2-27))	3 (1-9)
H-index	President	4 (2-9.5)	3 (1.5-9)
	Vice President	3 (1-34)	5 (2-15.5)
	Members	4 (2-11)	3 (2-7)
Citations	President	50.5 (11-472.5)	62 (11.5-270.5)
	Vice President	41 (3-3518)	52 (7-997.5)
	Members	77 19-49.5)	32 (10-137)

More males within FM societies were affiliated with a University 453 (47.19%) compared to 353 (36.77%) females. There were more males within each academic rank except at the Assistant Professor rank; see Table 1. These values were found to be statistically significant (Chi-square = 14.68, *p *= 0.012). When analyzing specific variables across increasing academic ranks, males held higher medians for publications, H-index, and citations for most academic ranks. Females held higher median for publications, H-index, and citations when they were found to have an affiliation, but no formal rank was found (“affiliated, but no rank found”).

Ordinal logistic regression

Generalized ordered logistic regression was used to estimate the impact of the primary predictors (sex, leadership ranks, academic ranks, publications, citations, h index, years since first publication, years of active research) on becoming a president in CFPC, RACGP, and WONCA. This model was selected instead of binary logistic regression because the outcome (Family Medicine Association category) had 3 ordered categories and the model failed the proportional odds assumption of standard ordered logistic regression. 

The regression model supports the decision to include the co-variants leadership ranks, academic ranks, publications, and citations. Gender even though was insignificant and was kept in the model, as it was the primary exposure of interest in the preliminary final model. The likelihood test indicated that it is statistically significant. Furthermore, its inclusion caused important changes in the coefficients for other variables in the model.

The final step in the model building process was an evaluation of interactions (cut off for interaction was 0.1). Interaction terms were created between each of the main effects in the model, one by one. There was a significant interaction between publications and years of active research (Z= - 1.38; p value= 0.17) in the models comparing them at all levels. When this interaction term is added to the model, the overall model improved. The log-likelihood increased.

Model 1

For category 0 (CFPC) as reference, against RACGP & WONCA (LR: Leadership Rank; AR: Academic Rank)

(g(x) = ln Pr = 1/ Pr = 0

g(x) = ˄β01+ ˄ β1(Females) + ˄β21 ( LR2) + ˄β22 (LR3) + ˄β31 (AR2) + ˄β32 (AR3) + ˄β33 ( AR4) + ˄β34 ( AR5) +˄β35 (AR6) + ˄β4 (Publications) + ˄β5 (Citations) +˄β6 (Publications * Years of Active Research)\)

Model 2

For category CFPC & RACGP as reference, against WONCA (LR: Leadership Rank; AR: Academic Rank)

g(x) = ln Pr = 2/ Pr = 0

g(x) = ˄β01+ ˄ β1(Females) + ˄β21 ( LR2) + ˄β22 (LR3) + ˄β31 (AR2) + ˄β32 (AR3) + ˄β33 ( AR4) + ˄β34 ( AR5) +˄β35 (AR6) + ˄β4 (Publications) + ˄β5 (Citations) +˄β6 (Publications * Years of Active Research)

The log odds of working in a first-in-command position within CFPC‘s associations, are 0.45 times for females as compared to male, adjusting for other variables. The log odds of working in a first-in-command position within WONCA’s associations are 1.23 times for females as compared to male, adjusting for other variables.

## Discussion

While the ratio of females to males is trending towards equal in medical school matriculants, there is a paucity of published literature on gender disparity within professional medical societies; however, it is reasonable to assume they follow similar trends to other medical specialties since these are closely associated [3,13-14].

This global perspective of FM societies shows that females comprise about 42% of the society committee workforce which is consistent with the number of females in the FM medical workforce, Canada (45%), Australia (42%), and the USA (38%) [5-6,10]. When compared with limited available literature of other medical societies, FM societies have a greater representation of females; neurosurgery (10%), endocrine and diabetes (39%) [3,13]. While the composition of females in FM societies has surpassed other medical societies, when comparing the research productivity within FM societies, males had a greater number of years of active research, years since their first publication, h-index, number of publications, and number of citations. Contributing reasons for this parallel those outlined by several other studies: work constraints, traditional gender role, social expectation, harassment, fewer same-sex mentors, fewer research grants awarded to females [3-4,15-17].

In addition to reasons outlined by other studies that contribute to decreased research productivity seen by females in medical societies, FM is also less research-intensive compared to other specialties; in Australia, there is 1 FM article per 1000 FM doctors, whereas there are 105/1000, 61/1000, and 148/1000 in internal medicine, surgery, and public health respectively [18]. Medical students who enter FM, do so with the intention of not entering research careers and focus more on patient care; in this study, we found that 59% of family physicians involved with societies had an H-index of 0 [18-19]. Other factors associated with decreased research productivity within FM are: lack of mentor support, decreased research activity within residency program, lack of time, lack of institutional support, additional responsibilities, and program size [20,21]. Most family physicians also tend to work in private offices and therefore are unable to compete with specialties that have the resources of a larger institution available to them; this global perspective found that 16% of family physicians had no academic affiliation. Even with academic affiliation and resources available, 80% of faculty in FM spent a half-day or less per week on research [22]; similarly, most physicians do not work within their academic affiliation full-time. They also have private offices in which they practice. In addition to the expected clinical and academic responsibilities, active members of FM societies have added responsibilities of their respective societies’ agenda [21]. This burden is compounded for females, younger physicians or those that are single parents [23-24].

In order to influence change, it is necessary to target individual and institutional policies. Despite WONCA being composed of representatives from countries of varying progressive attitudes, as of the writing of this manuscript, they are also the only society to have a “Women and Family Medicine” Working Group; part of this working group’s responsibilities is to advocate for women physicians. This helps create an environment that supports gender equity and is vital in the establishment of gender parity; women in WONCA had more publications and more years of active research compared to other societies and greater odds of holding a “1st in command” position. Similarly, providing support through research training dedicated to FM physicians can help increase research productivity; FM physicians who were provided training reported having increased skills in research methodologies, ability to publish, ability to obtain funding, increased academic promotions, institutional recognition, and improved ability to teach using evidence-based medicine [21]. 

According to AAMC recommendations, in order for an organization to have long term success, they should (summarized) [25]: 1) Emphasize faculty diversity; 2) target women's professional development needs within the context of helping all faculty maximize their faculty appointments; 3) assess which institutional practices tend to favor men's over women's professional development; 4) enhance the effectiveness of search committees to attract women candidates; 5) financially support institutional Women in Medicine programs and regularly monitor the representation of women at senior ranks. Similar recommendations have been made in Canada and Australia [26-27]. Greater effort, also, needs to be undertaken to determine which programs are most successful in supporting females and which programs aid the retention of females in FM, while also supporting research productivity within FM.

Limitations

Our study has its share of limitations including the reliance on publicly available self-reported data via society websites and professional websites such as LinkedIn that may skew against women; an internal survey of members, LinkedIn found that men skewed their profiles to highlight more senior-level experience and had longer profile summaries than women did [28]. It is, therefore, possible that achievements among the women in the cohort were underestimated, although it should be noted that most leadership information was obtained directly from official society and faculty websites.

This study also used Scopus to determine active years of research, which has certain limitations. First, Scopus does not include citations before 1995, which may skew the results for senior faculty. Second, because years of active research are determined by an author’s year of first publication, it is possible that those who take prolonged breaks during their careers may have their years of active research overestimated, a finding that likely disproportionately affects women, who generally have more familial obligations. Moreover, some individuals may have changed their last names after a change in marital status. In such a scenario, it is not possible to correlate their academic parameters including the number of publications or H-index before and after the change of name. Thus, there is no way to account for and mitigate this limitation in the present study.

## Conclusions

While FM societies have a greater representation of females compared to other medical societies, the existence of gender disparity parallels the reasons outlined in other studies. The caveat is that FM is not as research-intensive, compared to other medical specialties. In order to reach gender parity within FM societies and improve research productivity in FM, we recommend that societies begin with instituting a working group or committee that focuses on addressing and supporting the needs of women within FM. Greater efforts also need to be given to evaluate which programs have the greatest impact on reducing gender disparity at a multi-institutional level.
